# Lifestyle Interventions to Prevent Type 2 Diabetes: A Systematic Review of Economic Evaluation Studies

**DOI:** 10.1155/2016/2159890

**Published:** 2016-01-13

**Authors:** Koffi Alouki, Hélène Delisle, Clara Bermúdez-Tamayo, Mira Johri

**Affiliations:** ^1^TRANSNUT, WHO Collaborating Centre on Nutrition Changes and Development, Department of Nutrition, Faculty of Medicine, University of Montreal, 2405 Chemin de la Côte Sainte-Catherine, Montreal, QC, Canada H3T 1A8; ^2^Institut de Recherche en Santé Publique de l'Université de Montréal (IRSPUM), University of Montreal, 7101 Avenue du Parc, 3e Étage, Montréal, QC, Canada H3N 1X9; ^3^Centre de Recherche du Centre Hospitalier de l'Université de Montréal (CRCHUM), Tour Saint-Antoine, 850 Rue Saint-Denis, Montréal, QC, Canada H2X 0A9; ^4^Department of Health Administration, School of Public Health (ESPUM), Faculty of Medicine, University of Montreal, 7101 Avenue du Parc, 3e Étage, Montréal, QC, Canada H3N 1X9

## Abstract

*Objective*. To summarize key findings of economic evaluations of lifestyle interventions for the primary prevention of type 2 diabetes (T2D) in high-risk subjects. *Methods*. We conducted a systematic review of peer-reviewed original studies published since January 2009 in English, French, and Spanish. Eligible studies were identified through relevant databases including PubMed, Medline, National Health Services Economic Evaluation, CINHAL, EconLit, Web of sciences, EMBASE, and the Latin American and Caribbean Health Sciences Literature. Studies targeting obesity were also included. Data were extracted using a standardized method. The BMJ checklist was used to assess study quality. The heterogeneity of lifestyle interventions precluded a meta-analysis. *Results*. Overall, 20 studies were retained, including six focusing on obesity control. Seven were conducted within trials and 13 using modeling techniques. T2D prevention by physical activity or diet or both proved cost-effective according to accepted thresholds, except for five inconclusive studies, three on diabetes prevention and two on obesity control. Most studies exhibited limitations in reporting results, primarily with regard to generalizability and justification of selected sensitivity parameters. *Conclusion*. This confirms that lifestyle interventions for the primary prevention of diabetes are cost-effective. Such interventions should be further promoted as sound investment in the fight against diabetes.

## 1. Background

Noncommunicable diseases (NCDs) are steadily rising, affecting both developing and developed countries. This is a consequence not only of population aging, but also of the nutrition transition towards westernized diets and sedentary lifestyles. The nutrition transition is fueled by socioeconomic and technological development as well as globalization and accelerated urbanization [1]. Among the nutrition-related NCDs, diabetes is a major concern because its prevalence is rapidly increasing worldwide and particularly so in developing countries. Nearly 387 million people were affected in 2013. This number is expected to reach 592 million by 2035, with the Middle East, South East Asia, and Africa showing the fastest increase in the number of cases [2]. According to the International Diabetes Federation, 80% of people suffering from diabetes live in low- and middle-income countries. Diabetes is associated with several complications, leading to morbidity, disability, and premature mortality [2, 3]. Type 2 diabetes (T2D) is by far the most common form of the disease. Diabetes also entails a heavy economic burden for patients, households, and healthcare systems [4, 5].

T2D is a lifestyle disease, which can and should be prevented by intensive lifestyle interventions, characterized by changes in dietary habits and increased physical activity. Indeed, lifestyle interventions at the prediabetes stage have proved successful at reducing the incidence of T2D by 28.5% to 58%, in China (Da Qing), India (Indian Diabetes Prevention Program: IDPP-1), Finland (Diabetes Prevention Program, DPP), and the United States (Diabetes Prevention Program and Outcomes Study, DPPOS) [6–8]. Weight control is key to the prevention and management of diabetes independent of dietary composition [9]. As obesity is a major risk factor for T2D, lifestyle interventions aimed at weight loss or control are also critical to prevent T2D. Except for India and China, few studies have been conducted to date on diabetes prevention programs in low- and middle-income countries. In developed nations and even more so in low-resource countries, healthcare spending is a critical economic and political issue [10]. A recent World Health Organization report recommended addressing common lifestyle risk factors for NCDs, considering their cost-effectiveness, and their relative ease, and speed of implementation [11]. In resource-limited settings in particular, decision makers require information on the economic burden of NCDs, particularly T2D, and of the potential added value of lifestyle interventions for health and development. The economic evaluation of various preventative interventions is important in view of the urgent need for developing countries to set these NCDs as a public health priority, of the rapid increase in diabetes prevalence and of substantial variations in lifestyle intervention components and delivery.

There are limited systematic reviews on this topic and the most recent ones covered the period of 1985–2008 [12, 13]. Most economic evaluations of T2D prevention programmes pertained to developed countries partly owing to lack of relevant data in developing countries, while cost-effectiveness tends to be context-specific [14]. Our objective was to review economic evaluation studies of lifestyle interventions for the primary prevention of T2D and also for the control of obesity as key risk factor, based on data published since 2009. This review was intended to update knowledge on the cost-effectiveness of T2D prevention.

## 2. Methods

### 2.1. Search Process

In order to identify all relevant studies performing an economic evaluation of lifestyle interventions to prevent T2D and for obesity control, we searched the following databases: PubMed, Medline, the British National Health Services Economic Evaluation (NHS EES), CINHAL, Econ Lit, Web of sciences, EMBASE, and Latin American and Caribbean Health Sciences Literature (LILACS). We restricted our search to studies published in French, English, or Spanish between January 2009 and December 2014 as previous systematic reviews included studies published between 1995 and 2008. We used medical subject headings (MeSH) and other relevant terms to the topic as major constructs to build our search strategy. The MeSH or other relevant terms are related to economic, diabetes, and intervention constructs. To combine these, we used boolean operators “AND” and “OR” as appropriate. In addition, the reference lists of all included studies were scanned to identify any additional potentially relevant reports. For example, the PubMed search combined (i) “Cost-benefit-analysis (MeSH)” OR “Costs and cost-analysis (MeSH)” OR “Cost-benefit (title)” OR “Cost-effectiveness (title)” OR “Cost-utility (title)” OR “Economic evaluation (title)” AND (ii) “Type 2 diabetes (MeSH)” OR “Non Insulin dependent diabetes (MeSH)” OR “Gestational diabetes (MeSH)” OR “Obesity (MeSH)” OR “Impaired glucose tolerance (title)” OR “Prediabetes (title)” AND (iii) “Diet (MeSH)” OR “Physical activity (MeSH)” OR “Diet therapy (MeSH)” OR “Lifestyle (MeSH)” OR “Risk reduction behaviour (MeSH)” OR “Prevention (title)” OR “Lifestyle modification (title)” OR “Lifestyle advice programme (title)” OR “Non pharmacological prevention (title)”. Appendix shows the majors constructs used in our search strategy.

### 2.2. Study Selection

In order to select the relevant studies for this review, we screened titles and abstracts using a three-stage process. At the first stage, two authors (Koffi Alouki and Clara Bermúdez-Tamayo) independently selected studies based on abstracts and titles. They rejected clearly irrelevant titles, abstracts only, and duplicates. They cross-checked their results and retained candidate studies for full paper screening. Finally, studies were screened by reading the full papers. Through this process, the same coauthors resolved the disagreements. On the basis of full assessment and discussion of each study, they jointly selected the studies that were to be included in the review. This review was not blinded.

The study selection was guided by the following inclusion criteria:

- Original research articles published in peer-reviewed journals were candidates for inclusion.
- Type of economic evaluation: the selected studies conducted a full economic evaluation as defined by Drummond et al. [47]. “Full economic evaluations” are studies in which a comparison of two or more treatments or care alternatives is undertaken and in which both the costs and outcomes of the alternatives are examined in terms of cost-effectiveness, cost-utility, or cost-benefit analyses.
- The participants: the population groups targeted for the primary prevention of T2D were adult subjects (over 18 years old) who were at high risk of developing the disease because of obesity, impaired glucose tolerance, impaired fasting glycaemia, or gestational diabetes.
- The interventions: we considered dietary modifications or physical activity or both to prevent T2D or control obesity.
- The comparison: any comparison arm or group used against the lifestyle intervention was accepted for this review.
- Outcomes: these were cost per QALY (Quality Adjusted Life Years) gained, cost per life year gained, cost per DALY (Disability Adjusted Life Years) averted, cost per diabetes case averted, and other relevant outcomes.
- Studies published between January 2009 and December 2014.
- Studies that were published in English, French, or Spanish.

### 2.3. Data Extraction and Synthesis

The first reviewer (Koffi Alouki) extracted data using a standardized data extraction form built according to the Consolidated Health Economic Evaluation Statement (CHEERS) [15]. The second reviewer (Clara Bermúdez-Tamayo) checked that the extracts and discrepancies were resolved through discussion. A third reviewer was not necessary as all queries were resolved by consensus. Data extracted included the type of economic evaluation, subjects' characteristics (e.g., age, biological and anthropometric characteristics), intervention details (e.g., duration, location, intensity, and mode of delivery of the intervention), comparator, analytical model used, effectiveness data, sensitivity analysis, and reported outcomes relevant to the review. Fields extracted are summarised in Table 1. There are some trial-based type studies while others relied on model-based studies or previous trial results to extrapolate by using modeling techniques, something which was previously highlighted in the literature [10]. The interventions compared, the population groups targeted, and the outcomes reported also varied across studies. For these reasons we chose a narrative approach for this systematic review, as is usually done for systematic reviews of economic evaluations.

### 2.4. Assessment of Study Quality

We used the British Medical Journal (BMJ) quality assessment checklist, a 36-item scale, to assess the quality of the studies [36]. This checklist was developed with the aim of standardizing the presentation of study data, thereby contributing to the quality of economic evaluations. We also assessed any risk of bias due to conflicts of interest or sponsorship of studies. Each item was answered by “No” or “Yes” or “Not applicable.” We gave a score of 0 if the answer was “Yes” and 1 if it was “No.” Then we summed up the number of “No” responses to obtain a global score in which a higher score represented poorer quality. Two reviewers (Koffi Alouki, Clara Bermúdez-Tamayo) conducted this operation independently and disagreements were resolved through discussion. Quality rating was used to interpret the results but no study was excluded on this basis.

## 3. Results

### 3.1. Overview of Studies

**Figure 1:**
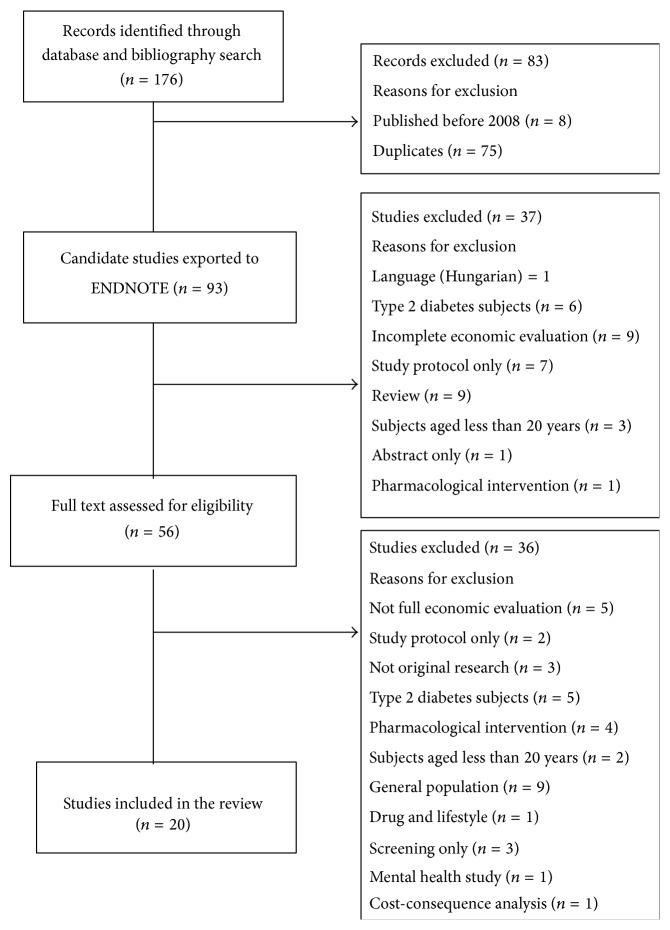
Flowchart of overall systematic search process.

The stages of the search process are illustrated in the flowchart of Figure 1. The search yielded 176 abstracts. After reviewing the abstracts, subsequent reference tracking, and excluding duplicate articles, we narrowed the focus to 56 candidate studies having performed an original economic evaluation. Further review of the full text resulted in 20 studies that met our inclusion criteria.  Table 2 summarizes the retained study characteristics and the analytical approach used. The studies were conducted in the UK, USA, Canada, Australia, Germany, Finland, the Netherlands, Singapore, Sweden, and China. Three studies performed only a cost-effectiveness analysis while 13 studies assessed only cost-utility. The four remaining studies combined cost-effectiveness and cost-utility analysis. There were 10 studies using a Markov-type model or a decision tree, or both, to make projections of the evolution of T2D [22–24, 26–29, 31, 33, 35]. Seven studies performed trial-based analyses [16–21, 30]. The period for which the potential benefit of the intervention was simulated ranged from three years to lifetime. The two studies targeting women with gestational diabetes were conducted throughout pregnancy [20, 31]. In eight studies [16, 17, 22, 23, 25, 27–29], data on efficacy and effectiveness used in simulations were drawn from the major randomized controlled trials on T2D prevention: DPPOS [37], DPP [38], and Da Qing [39]. In a further six studies, effectiveness data was based on synthesis of multiple studies [24, 31–35]. Six of the 20 studies used effectiveness data from a specific intervention conducted in the same country where the economic evaluation was carried out [18–21, 26, 30]. The items included in the cost calculations were obviously dependent on the study perspective. The cost to the healthcare system only or to the whole society was generally considered. Only two studies adopted a third payer perspective. The costs and the effectiveness were discounted using rates varying from 3% to 5% according to the countries. To test the robustness of the results, a sensitivity analysis was performed in most studies. It was not possible to identify the source of funding for only one study [27]. Among the other 19 studies, four had not reported any funding. Most studies (16/20) were funded by public agencies.

### 3.2. Description of Interventions

The interventions included in this review varied from the simple provision of information to active behaviour change schemes. The lifestyle changes described across studies pertained to diet, physical activity, or both. In some cases, the screening of subjects at high risk preceded the interventions without explicitly taking account of the screening in the cost calculations [17, 18, 24]. Of 10 model-based studies, seven simulated interventions based on the DPP [23–25, 28, 29] or the China Da Qing study [22, 27]. The DPP intervention goal was to achieve and maintain a weight reduction of at least 7% of initial body weight through diet and physical activity of moderate intensity, such as brisk walking for at least 150 minutes per week. The DPP program included a lifestyle curriculum, taught by case managers on a one-to-one basis during the first 24 weeks after enrollment. The teaching was flexible, culturally sensitive, and individualized. Subsequent individual sessions (usually monthly) and group sessions with the case managers were designed to reinforce the behavioral changes. The aim of nutritional counselling was to help the participants achieve a diet containing 10% of total energy intake as saturated fats, 5–10% as polyunsaturated fats, 25–30% as total fat (saturated, monounsaturated, polyunsaturated, and trans fatty acids), and 25 to 35 grams of fibre per day. One study described a commercial program consisting of a low-calorie diet and physical activity advice [31]. One study carried out the economic evaluation of the “DASH” diet (Dietary Approaches to Stop Hypertension) or a “low fat” diet [33] to reduce the disease burden related to excess body weight. The “DASH” diet emphasizes reduced consumption of fat, red meat, sweets, and sugar-containing beverages, and the program recommends 180 minutes per week of moderate intensity physical activity. Individual or group sessions were held every 4 to 8 weeks. One study performed the economic analysis of lifestyle changes achieved through e-learning devices [32]. Another economic evaluation from the United Kingdom was conducted on a program focusing on very low-calorie diets [34]. Two studies reported on the economic evaluation of interventions consisting solely of physical activity [21, 35]. The first one was based on exercise referral schemes [40]. It took the form of a structured programme of exercise in a fitness centre and incorporating monitoring of individual performance. The second study pertained to sessions of physical activity that included 60 minutes of walking in addition to exercise. The exercise sessions consisted of aerobic and strength exercises. Depending on the studies, the comparison (or control) interventions were common standard care, placebo, metformin (850 mg twice daily), or simple advice in writing for physical activity and nutrition.

### 3.3. Analysis of Costs

Reported costs depended on the chosen perspective, the nature of the intervention, the target population, and the time horizon. The various costs are shown in Table 2. There is no consistency across studies in the items included to estimate the costs of interventions. All studies considered direct costs including medical costs [16, 17, 20–23, 25, 30]. Medical records were the most common source for data on diabetes and complications. Generally, the direct medical costs were copayment fees for treatment, diagnostic testing, prescription drugs, and medical supplies. The direct costs also included the costs of visits to healthcare providers and exercise physiologist and metformin cost for controls. Direct nonmedical costs pertained to services such as transportation of subjects and family members to clinics, as well as special food in some instances. Lost income for the patients and their families and the costs of hiring nurses or care providers were recorded as indirect costs. Four studies [18, 20, 21, 23] reported on the estimated cost of productivity loss. In four of the 20 studies [22, 24, 25, 29], cost estimates were only presented as category totals, without breakdown into individual items and without a separate presentation of the resources needed for the interventions. Two studies described the unit costs of various resources, using the ingredient approach [27, 28]. Physical quantities of necessary inputs were counted and multiplied by unit prices to obtain total costs [41]. Pricing sources were reported in all studies. For most studies, price data came from healthcare facilities, from patient records, or else from estimates based on published data. Other costs such as transport were self-reported by the subjects. Seven studies recorded costs alongside the trials [17–19, 21]. Except for four studies [18–21], all studies discounted the costs by 3% to 5%.

### 3.4. Effectiveness Data

The effectiveness data were usually derived from major randomized controlled trials on T2D prevention [7, 17, 19, 38, 39]. Nearly one-third of studies estimated effectiveness based on meta-analyses or literature reviews [24, 31–35]. More than half the studies expressed intervention benefits in terms of QALYs gained. Three out of the 20 studies reported effectiveness as DALYs averted [24, 31, 33]. Diabetes cases averted by the interventions were reported in two studies [19, 24]. Another study estimated the delay in progression to T2D attributable to the lifestyle intervention [28]. One study reported change in utility as an outcome measure, based on the 15-dimension questionnaire tool (15D) to assess quality of life but without conversion to quality adjusted life years [20]. Several tools were used to assess the effects of interventions. Three studies used disability weights. Nine studies (9/20) used the EQ5D (EuroQoL 5-Dimension tool), three studies used the QWB-SA (Quality of Well Being-Self-Administered), two studies used the HRQoL (Health Related Quality of Life) system, one study used the VSA (Visual Scale Analog), and one study used the 15D or SF-36 (36-item short form survey instrument). Long-term assumptions about effectiveness were dissimilar. In some cases, the key assumption was that the expected effects of the intervention were for the short term with a linear decrease in effectiveness following the intervention [27]. Another study projected the same effectiveness during the whole duration of the time horizon projected. The probability of progressing from prediabetes to T2D in all studies using modeling techniques was always lower in the intervention arm consisting of lifestyle intervention than in the comparison arm, whether placebo, usual care, or metformin treatment. Assumptions regarding the effectiveness of screening were needed when screening was required before implementing interventions. The studies reported sensitivity and specificity of screening ranging from 75% to 100% [22, 25].

### 3.5. Cost-Effectiveness of Interventions

Cost-effectiveness results are presented in Table 2. Compared with usual care, placebo, or metformin, interventions based on lifestyle modifications were reported as cost-effective in 15 of 20 studies [16, 17, 19, 22–30, 33–35]. The conclusions are usually based on the incremental cost-effectiveness ratio as applied in the study countries. One study considered the WHO threshold to decide on the cost-effectiveness [23], according to which intervention is considered highly cost-effective when the ICER is below the GDP (Gross Domestic Product) per capita and cost-effective when the ICER ranges from 1 to 3 times GDP per capita. When a strategy improves health outcomes at lower cost, it is considered to be dominant and it is obviously preferred as cost-effective. Those interventions that are less effective and more costly (considered as dominated interventions) or more costly and more effective but with a resulting more expensive ICER would unlikely be cost-effective; these studies were considered inconclusive. Three studies revealed that the interventions were dominant [17, 22, 27]. However, the results reported in four studies were less favourable. Oostdam et al. [21] reported that a lifestyle intervention implemented during 32 weeks and targeting at-risk pregnant women in Germany was not cost-effective. Similar results were also reported in another intensive lifestyle intervention targeting at-risk pregnant women [20]. One study reported that a lifestyle intervention offered by nurses was not more cost-effective in reducing T2D risk than the control intervention consisting in the provision of a general health brochure [18]. Johansson et al. [26] examined the cost-effectiveness of lifestyle by sex and concluded that intervention was only cost-effective among women.

Sensitivity analysis, which allows assessing the reliability and the generalizability of the results [42], was performed in all studies except one [34]. Over half the studies performed univariate sensitivity analysis and eight studies performed bivariate sensitivity analysis. Probabilistic sensitivity analysis was used in ten studies [18, 21, 24, 26–29, 31, 32, 35]. The input parameters that were analyzed through a range of assumed values were discounted cost and outcomes (QALYs gained), variations of intervention cost, variation of probability of transition between disease states considered, the duration of interventions, the size of the population at risk, and performance and frequency of screening test. Although the ICER was sensitive to changes in the above parameters, it remained acceptable for most studies, except for the three studies reporting that the intervention was not cost-effective [20, 27, 31].

### 3.6. Quality Assessment

**Figure 2:**
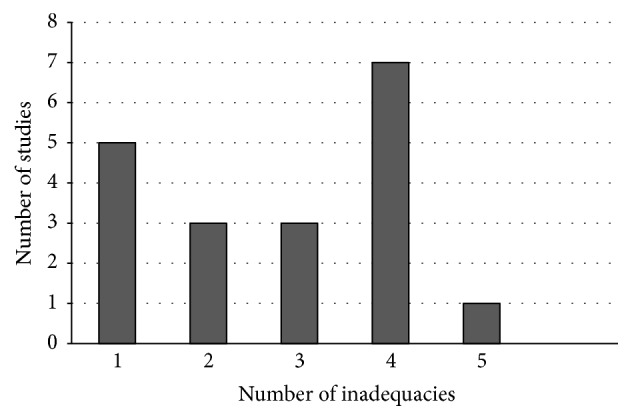
Limitations of studies as result of quality assessment.

Based on the BMJ checklist, the studies included in this review showed quality limitations in reporting as shown in Figure 2. The number of such limitations varied from 1 to 5. Only one study [19] had no methodological shortfall. Out of the remaining 20 studies, 15 presented at least two methodological limitations and five studies showed only one. More than half (12/20) of studies did not address the issue of generalizability. The justification of the parameters used for the sensitivity analysis was presented by only few studies. The models of five studies were not fully described or their choice was not justified. Three studies did not explicitly declare potential conflicts of interest. Eight studies did not report separately the resources for the interventions and their unit cost.

## 4. Discussion

The purpose of this systematic review was to describe economic evaluations of lifestyle interventions to prevent T2D or control obesity in high-risk population groups and which were carried out since 2009. Our review was focused exclusively on interventions targeting at-risk adult subjects. Lifestyle interventions in communities and schools for the primordial prevention of T2D were excluded as a recent review reported on their cost-effectiveness [13]. We identified 20 studies, mostly conducted in developed countries. Our results confirmed those of former reviews [12, 13], which concluded that lifestyle interventions through physical activity or diet or combining both were generally cost-effective, with a few exceptions. In our review, five out of the 20 studies were inconclusive. There was a trend for a higher proportion of interventions targeting the prevention of T2D compared to those focusing on obesity to be cost-effective (11/14 versus 4/6, resp.). The inconclusive studies [18, 20, 21, 31, 32] included one on the prevention of T2D, two on the prevention of gestational diabetes among pregnant women, and two on obesity control. These studies were more costly with less effectiveness or more costly with higher effectiveness but resulting in a more expensive incremental cost-effectiveness ratio compared with standard care (or control treatment). Three of the seven studies conducted within trial were nonconclusive compared with three of the 13 studies based on models. This would suggest that model-based studies tend to overestimate cost-effectiveness. The short duration of some studies may also explain the absence of significant changes in the outcomes. Another inconclusive study pertained to an e-based intervention, which may not induce enough motivation for change. Overall, there was considerable heterogeneity in the nature of lifestyle interventions across studies, which hampered comparisons and possibly contributed to the inconsistencies in outcome findings. Study results could be influenced by intervention components, selection of participants, and methodological and modeling choices [43]. Additionally, thresholds of cost-effectiveness varied across countries and studies.

### 4.1. Which Outcomes, Which Costs, and Which Time Horizon?

Few studies evaluated effectiveness in terms of QALY although it is known that diabetes is associated with deterioration of the quality of life. QALY is a relevant parameter that allows comparisons of the burden of disease in terms of quality and quantity [44]. Most studies of this review used EQ-5D and few used HRQoL and SF-36. Preference-based health state classification systems were preferred in most studies to objective methods of evaluation of health states. McDonough and Tosteson [45] showed that, among studies that compared alternative preference-based systems, the EQ-5D tended to provide larger change scores and therefore more favourable results than the Health Utility Index (HUI), while the SF-6D provided smaller change scores and therefore less favourable ratios than the other systems. Hence, the choice of outcome as a measure of effectiveness has an impact on reported results. All studies reported costs in local currency and prices. Cost variations were due primarily to the chosen perspective (societal/health system or third party), to the various inputs for the interventions, and to differences of care unit costs across countries. The lifestyle interventions involved several health professionals, which explains the higher costs of these interventions compared to controls. In some but not all simulation studies, the costs of health infrastructure and training of personnel were also provided. Such inclusive costs would be particularly useful for low-resource countries where health systems are ill-prepared to tackle a chronic disease like T2D [46]. Few studies complied with the recommendation to present quantity and unit cost of inputs separately from total cost as advocated in the guidelines [36], resulting in lack of the information required for the replication of the intervention in other settings. Additionally, the reported costs were often incomplete. For instance, if a societal perspective is adopted, the costs related to lost productivity, premature mortality, and permanent and temporary disability should be computed which is not always the case. As stated in the guidelines [47, 48], the future costs beyond one-year time should be discounted, which was done in most studies and using different discount rates. Some interventions did not use discounting due to their short duration. Although the selected rates were not justified in most studies, they probably reflect the fact that there are no universally accepted discount rates for economic evaluations studies [47]. The time horizon should be long enough to capture any significant difference between the intervention and the comparison groups in terms of costs and outcomes [48]. Yet, it appeared too short in some studies to appraise and capture long-term impact of interventions. Particularly, in the management of obesity or gestational diabetes, the inconclusive results may be partly ascribed to too short interventions to capture the long-term benefits. The costs for treating T2D complications are onerous, and the temporal horizon of some studies based on model assumptions cannot guarantee that the complications will not appear later on. The conclusions regarding cost-effectiveness should therefore be taken with caution. Weight loss is also an objective of T2D prevention interventions. However, without sustained compliance of subjects with advocated lifestyle changes, weight regain over the long-term can alter the quality of life and impinge on the estimated cost-effectiveness of interventions. Considering exclusively the short-term effects of obesity control interventions can indeed be misleading [49]. For this reason, it is not impossible that the cost-effectiveness of some interventions reviewed here and focusing on obesity was overestimated.

Trial-based studies limited the results of interventions to health gains over the course of the intervention. In contrast, studies supported by models were based on assumptions on a longer temporal horizon. This approach, however, would omit taking into account other relevant events that may occur later on that will have an impact on the costs and on the quality of life or other outcomes [10]. Additionally, the nature of the model inevitably has a bearing on the estimated cost-effectiveness of the interventions.

### 4.2. What about T2D Modeling?

The analytical models varied across studies. Modeling diabetes evolution showed variations among studies with regard to the shift from normal glycaemia to prediabetes and then to diabetes and finally to the complications. With the exception of one study, the models however seemed to be consistent with the natural evolution of the disease as described in the literature [50]. The accuracy of the models is one of the criteria for sound economic evaluations, and it reflects the ability to capture the evolution of the pathology in a real situation [51, 52]. Yet, in some studies, the models showed complication states without disclosing the nature of the complications. Another limitation of most models is that complications are taken separately into account while subjects living with T2D can have several complications. Some studies [23, 25] did not consider the likelihood of glycaemia returning to normal in subjects with prediabetes. The simplification of analytical models may not help to consider these subtleties during the course of evolution of the disease in a real situation.

Most simulated interventions were based on epidemiological studies of T2D prevention that provide the reference evidence for lifestyle interventions as relevant strategy to prevent diabetes. The randomized controlled trials that measured the long-term effects in real life situations after cessation of the active intervention [37, 39] therefore provide important new data for the simulation studies to better estimate the long-term effectiveness of interventions to prevent diabetes. However, the efficacy observed in controlled experimental conditions is different from the expected effectiveness in the real world because of subject selection, recruitment, and follow-up and other factors that have a bearing on economic outcomes [10, 53]. Moreover, in some model-based studies, the at-risk subjects may not accurately match the at-risk subjects of the original studies, so that the assumed effectiveness in these studies is uncertain. The effectiveness of interventions in the real world often falls a long way short of the maximum efficacy shown in trials [54]. For instance, a retrospective observational analysis of overweight and obese subjects demonstrated that, compared with the 58% reduction in risk of progression to diabetes seen in the DPP trial, risk reduction for incident diabetes in subjects who participated in the study in an intense and sustained way was lower [55]. The effectiveness was also shown to vary according to the setting of the intervention [56].

### 4.3. Quality of Studies

Assessment of quality of studies revealed methodological shortcomings. Most articles lacked one or several reporting items of the BMJ checklist for quality of economic evaluation studies although this checklist was issued before these studies were conducted. Another limitation observed is the lack of a clear description of the models [34], while the guidelines recommend transparency in their description [47]. In some cases, the models did not adequately capture the natural history of the disease, leading to questionable conclusions. Conversely, in another study [27], the model did not include the complication state, which would have been relevant in the natural history of diabetes. However, although we performed an evaluation of the quality of studies, we deliberately chose not to exclude any study on that basis. In any case, excluding the poorer quality studies would not have altered the conclusions.

### 4.4. Strengths and Weaknesses of the Review

We used a comprehensive electronic search strategy using preestablished criteria in common medical literature databases. As all studies are not referenced in electronic databases, we revisited the bibliography of each selected study to ensure that our search was exhaustive. Two reviewers cross-checked the database to identify the relevant studies. At variance with previous reviews on the economic evaluation of T2D prevention studies, ours also included intervention studies targeting obesity as main risk factor for T2D and not only the interventions aiming directly at T2D prevention. Our review also updates the results of these former reviews. Ours also encompassed the assessment of study quality based on the BMJ guidelines designed for the critical appraisal of economic evaluation studies. We adopted a dichotomous scale for each item, however, which does not reflect the level of completeness of the information reported for each item considered. It is also recognized that limiting the candidate studies to those published in French, English, and Spanish is a potential source of publication bias. Due to the heterogeneity in the methods and results of the different studies, we were not able to perform a meta-analysis, which is usually considered the “gold standard” but which is not often feasible with economic evaluation studies.

## 5. Conclusion

The present review on the cost-effectiveness of lifestyle modification interventions showed, with only a few exceptions, that these interventions targeting adult subjects at high risk for diabetes were cost-effective despite different assumptions regarding disease progression and variations in the delivery of these interventions. The results are consistent with conclusions of former reviews, confirming the importance of lifestyle interventions combining diet and physical activity to prevent diabetes in at-risk population groups. This review also broke new ground by assessing the methodological limitations of the economic evaluations and the quality of reporting, to aid in interpretation of results. Lifestyle interventions should be further stressed as an effective strategy to prevent or delay diabetes. Unfortunately, few studies have been conducted in resource-poor countries in spite of a dire need for such data, and the findings from developed countries are not entirely relevant. Future research should address the effectiveness and cost-effectiveness of such interventions in low-income country settings, where the prevalence of T2D is soaring. Meanwhile, the data of the present review provide compelling arguments for policy makers to implement measures to prevent T2D.

## Figures and Tables

**Table 1:** General features of selected studies.

Study	Country	Population	Intervention	Variables of interest	Comparison	Time horizon	Analytical approach	Study design
*For diabetes prevention*								

Herman et al. [16], 2012	USA	≥25 y.o. IGT/IFG, BMI ≥ 24 (≥22 for Asians)	DPP lifestyle modification	Diabetes cases prevented, QALYs	Metformin, placebo	10 years	Trial-based study	CU

Herman et al. [17], 2013	USA	≥25 y.o. IGT/IFG, BMI ≥ 24 (≥22 for Asian)	Lifestyle modification and metformin	Diabetes cases prevented, QALYs	Placebo	10 years	Trial-based study	CU

van Wier et al. [18], 2013	Netherlands	Adults aged 30–50 y at risk of T2D	Lifestyle intervention implemented in primary care	Risk of T2D, risk of CVD, and CVD mortality in the following 10 years	Provision of health brochures	10 years (duration 2 years)	Trial-based study	CU/CE

Sagarra et al. [19], 2014	Spain	Adults aged 45–75 y with IFG/IGT	Lifestyle intervention (individual or group intensive intervention)	Diabetes cases prevented, QALYs	Routine care	4 years	Trial-based study	CU/CE

Kolu et al. [20], 2013	Finland	≥40 years BMI ≥ 25 or IGT, history of macrosomia, and type 2 or type 1 diabetes in first- or second-degree relatives	Lifestyle modification	Health perception, birth weight, and quality of life	Routine care	37 weeks	Trial-based study	CU/CE

Oostdam et al. [21], 2012	Germany	Overweight pregnant women and at least one of the following: history of macrosomia, GDM, or first grade relative with diabetes or obese	Exercise program (FitFor2)	Maternal fasting blood glucose, QALYs, infant birth weight, and insulin sensitivity	Routine care	32 weeks	Trial-based study	CU

Liu et al. [22], 2013	China	Age ≥25 y, IGT	One-time screening for IGT/T2D with positive case receiving (i) lifestyle intervention/diet; (ii) lifestyle intervention/exercise; (iii) both diet and exercise; (iv) one-time screening alone.	Remaining survival years and QALYs	Control	40 years	Model-based study (decision tree and Markov)	CU

Png et al. [23], 2014	Singapore	Subjects with prediabetes (IFG/IGT)	Lifestyle modification	QALYs	Metformin/ placebo	3 years	Model-based study (decision tree)	CU

Bertram et al. [24], 2010	Australia	Age ≥45 y and high BMI, family history of T2D, or people from indigenous, and women with GDM	Diet and/or exercise,	Diabetes cases prevented, DALYs Averted	Acarbose, metformin, and orlistat	Lifetime	Model-based study (Markov)	CE

Mortaz et al. [25], 2012	Canada	Age ≥40 y and first-degree relative with T2D, high risk population groups (aboriginals, Hispanics, Asians, or Africans), and history of IGT/IFG, GDM, hypertension, dyslipidemia, overweight, abdominal obesity, and polycystic ovary	Screening followed by lifestyle intervention	QALYs	No screening	10 years/ lifetime	Model-based study (Markov)	CU

Johansson et al. [26], 2009	Sweden	Age 30–56 y and at risk of chronic disease without known diabetes	Lifestyle intervention	QALYs	Routine care	10 years	Model-based study (Markov)	CU

Neumann et al. [27], 2011	Germany	Subjects at high risk of developing T2D	Lifestyle intervention	QALYs	Routine care	Lifetime	Model-based study (Markov)	CU

Palmer and Tucker [28], 2012	Australia	Mean age 50.6 y with IGT/IFG, BMI ≥ 34	Intensive lifestyle intervention, Metformin	QALYs	Control	Lifetime	Model-based study (Markov)	CU

Smith et al. [29], 2010	United States	BMI ≥ 25 and the 4 components of MetS as defined by NCEP/ATP III	Lifestyle intervention	QALYs	Routine care	3 years	Model-based study (Markov)	CU

*For obesity control*								

Tsai et al. [30], 2013	USA	BMI 30–50, plus abdominal obesity plus at least one of the 4 other MetS criteria	Brief lifestyle counselling	QALYs and kilograms lost per year	Routine care	2 years	Trial-based study	CU/CE

Cobiac et al. [31], 2010	Australia	Age ≥ 40 y and BMI ≥ 27	“Lighten up to Healthy Lifestyle” and “Weight Watchers”	Weight lost/DALYs averted	Routine care	12 months	Model-based study (Markov)	CE

Miners et al. [32], 2012	United Kingdom	Age ≥ 50 y and BMI ≥ 30	E- learning devices to promote healthy diet and physical activity	Weight lost/QALYs gained	Routine care	Lifetime	Model-based study (e-learning economic evaluation model)	CU

Forster et al. [33], 2011	Australia	Age ≥ 40 y and BMI ≥ 25	The Dietary Approach to Stop Hypertension (DASH) and low fat diet intervention	Weight lost/DALYs Averted	Routine care	100 years	Model-based study (Markov)	CE

Lewis et al. [34], 2014	UK	Adult subjects with BMI ≥ 30	Lighter Life total (a very low calorie diet total dietary replacement) weight reduction program and group support appropriate for obese people	Weight lost, QALYs gained	(A) With BMI ≥ 30 group: (1) no treatment, (2) lifestyle intervention, (3) weight watchers, (4) slimming world, and (5) lighter life total movement only (B) With BMI ≥ 40 group: (1) no treatment, (2) gastric banding, (3) gastric bypass, and (4) lighter life total movement only	10 years	Not specified	CU

Anokye et al. [35], 2011	United Kingdom	Age 40–60 y, sedentary lifestyle	Exercise Referral scheme in physical activity	QALYs	Routine care	Lifetime	Model-based study (decision tree)	CU

BMI: body mass index; CE: cost-effectiveness; CU: cost-utility; CVD: cardiovascular disease; DPP: diabetes prevention program; GDM: gestational diabetes mellitus; IGT/IFG: impaired glucose tolerance/impaired fasting glucose; T2D: type 2 diabetes.

**Table 2:** Economic evaluation details of studies.

Study	Currency, discount rate	Perspective	Costs	Effectiveness measure	Incremental cost-effectiveness ratio	Is intervention cost-effective? (benchmark)^*∗∗∗*^
*For diabetes preventions *						

Herman et al. [16], 2012	US$, 2010, 3%	Health system and societal	Direct medical and nonmedical costs + intervention costs	QALYs	Lifestyle compared to placebo, health system perspective: 12,878$US/QALY; societal perspective: 23,597$US/QALY	Yes

Herman et al. [17], 2013	US$, 2010, 3%	Health system and societal	Direct medical and nonmedical costs + intervention costs	QALYs	(a) Health system perspective: cost saving (lifestyle versus placebo) cost saving (metformin versus placebo); (b) societal perspective: the ICER was 3,235$US/QALY (lifestyle versus placebo)	Yes

van Wier et al. [18], 2013	Euros, 2008	Societal	Intervention costs + productivity lost costs	QALYs, 9-year risk of developing T2D	−50,273€/QALY gained; the ICER of 9-year risk for developing T2D was −1416€ Lifestyle guidance offered by practice nurses was not more effective in reducing these risks than the provision of general health brochures	No

Sagarra et al. [19], 2014	Euros, 2007	Health system	Intervention costs	Diabetes cases prevented and QALYs	376.17€/case of T2D averted; 3243€/QALY gained	Yes

Kolu et al. [20], 2013	Euros, 2009	Societal	Direct medical costs + lost productivity costs + health care intervention costs	Health perceptions (visual analog scale), birth weight, 15D (quality of life)	Each gram of birth weight prevented requires an additional cost of €7; each perceived health gain requires additional cost of 1697€	No

Oostdam et al. [21], 2012	Euros, 2009	Societal	Direct and indirect costs	Maternal fasting blood glucose, QALYs gained, infant birth weight, and insulin sensitivity	Being not cost-effective versus control group for blood glucose, insulin sensitivity, infant birth weight, and QALYs gained	No

Liu et al. [22], 2013	US$, 2007, 3%	Societal	Direct and nonmedical costs, indirect costs	QALYs	Savings: US$ 2017 per subject	Yes

Png et al. [23], 2014	US$, 2012, 3%	Health system and societal	Direct medical costs, direct nonmedical costs, and indirect costs	QALYs	Health system perspective: US$ 17,184/QALY for lifestyle modification versus placebo; societal perspective: US$ 36,367/QALY	Yes (WHO benchmark)

Bertram et al. [24], 2010	AU$, 2010, 3%	Health system	Directs cost of each intervention	DALYs averted, diabetes cases averted	AU$ 23.000/DALY averted (diet and exercise); AU$ 22.000/DALY averted (metformin)	Yes

Mortaz et al. [25], 2012	CAN$, 2010, 3%	Health system	Direct cost per person	QALYs	Conventional screening every 3 years was more effective over no screening	Yes

Johansson et al. [26], 2009	Krona, 2004, 3%	Societal	The societal costs	QALYs	For women QALY losses were lower and cost increases were lower; among men, the net costs were larger and QALYs lost were higher in all three treatments than in controls	Yes for women, No for men

Neumann et al. [27], 2011	Euros, 2007, 3%	Societal	Direct cost + interventions cost	QALYs	The ICERs were negative, for men and women who started the intervention when aged 30–50 years	Yes

Palmer and Tucker [28], 2012	AU$, 2009, 5%	Third-party payer and health system	Direct medical costs + intervention costs	QALYs	Intensive lifestyle change was cost-effective compared to controls	Yes

Smith et al. [29], 2010	US$, 2000, 3%	Societal	Direct costs + interventions costs	QALYs	$ 3,420/QALY due to decrease in diabetes incidence with intervention	Yes

*For obesity control *						

Tsai et al. [30], 2013	US$, 2010	Health system	Intervention costs + health care providers + medication	QALYs	$US 3134/QALY (BLC compared to usual care) $US 115397/QALY (EBLC compared to routine care)	Yes

Cobiac et al. [31], 2010	US$, 2003, 3%	Health system	Direct and intervention costs	DALYs averted	Both weight loss programmes produced small improvements in the exposed subjects compared to current practices	No

Miners et al. [32], 2012	£UK, 2009, 3,5%	Health system	Direct and intervention costs	QALYs	The lowest was 102,000£/QALY; however, scenario contains women associated with lower QALYs compared with men	No

Forster et al. [33], 2011	AUS$, 2003, 3%	Health system	The intervention + direct costs related to each state in the model	DALYs averted	AUS$ 12000/DALY averted (DASH diet) AUS$ 13000/DALY averted (low fat diet)	Yes

Lewis et al. [34], 2014	£UK, 2012, 3,5%	Health system	Intervention costs	QALYs	For subjects with BMI ≥30, lighter life is cost-effective; for subjects with BMI ≥40 eligible for bariatric surgery, gastric bypass is cost-effective	Yes

Anokye et al. [35], 2011	£UK, 2011, 3,5%	Third-party payer	Direct costs + intervention costs	QALYs	20,876£/QALY	Yes

BLC: brief lifestyle counselling; DALY: Disability Adjusted Life Year; DASH: dietary approach to stop hypertension; EBLC: enhanced brief lifestyle counselling; ICER: incremental cost-effectiveness ratio; MetS: metabolic syndrome; QALY: Quality Adjusted Life Year; VAS: visual analog scale; 15D: 15-Dimension.

^*∗∗∗*^According to authors conclusions about the value of one or more interventions to control obesity or prevent type 2 diabetes. One study used WHO benchmark to justify the conclusion as mentioned in bracket.

**Table 3:** List of combinations of terms used for research studies in the database.

Economic concepts	Type 2 diabetes concepts	Intervention concepts
*MeSH terms*		
Cost-benefit analysis Cost and cost analysis	Type 2 diabetes mellitus Noninsulin dependent diabetes mellitus Gestational diabetes	Diet Physical activity Diet therapy Lifestyle Risk reduction behaviour

*Title terms*		
Cost-effectiveness Cost-utility Economic outcomes Cost outcomes Economic evaluation Cost	Impaired glucose tolerance Prediabetes	Prevention Lifestyle modification Nonpharmacological prevention Primary prevention Nutritional intervention Dietary intervention Nutrition counselling Prevention programme Lifestyle advice programme

## Appendix

See Table 3.

## References

[1] Popkin B. M. (2006). Global nutrition dynamics: the world is shifting rapidly toward a diet linked with noncommunicable diseases. *American Journal of Clinical Nutrition*.

[2] IDF (2014). *International Diabetes Federation Diabetes Atlas*.

[3] Mbanya J. C. N., Motala A. A., Sobngwi E., Assah F. K., Enoru S. T. (2010). Diabetes in sub-Saharan Africa. *The Lancet*.

[4] Kirigia J. M., Sambo H. B., Sambo L. G., Barry S. P. (2009). Economic burden of diabetes mellitus in the WHO African region. *BMC International Health and Human Rights*.

[5] Ankotche A., Binan Y., Leye A. (2009). Graves conséquences du coût financier du diabète sur sa prise en charge, en dehors des complications, en Afrique sub-saharienne : l’exemple de la Côte-d’Ivoire. *Médecine des Maladies Métaboliques*.

[6] Gong Q., Gregg E. W., Wang J. (2011). Long-term effects of a randomised trial of a 6-year lifestyle intervention in impaired glucose tolerance on diabetes-related microvascular complications: the China da Qing Diabetes Prevention Outcome Study. *Diabetologia*.

[7] Ramachandran A., Snehalatha C., Mary S., Mukesh B., Bhaskar A. D., Vijay V. (2006). The Indian Diabetes Prevention Programme shows that lifestyle modification and metformin prevent type 2 diabetes in Asian Indian subjects with impaired glucose tolerance (IDPP-1). *Diabetologia*.

[8] Orchard T. J., Temprosa M., Barrett-Connor E. (2013). Long-term effects of the diabetes prevention program interventions on cardiovascular risk factors: a report from the DPP Outcomes Study. *Diabetic Medicine*.

[9] Islam S. M., Purnat T. D., Phuong N. T., Mwingira U., Schacht K., Fröschl G. (2014). Non-Communicable Diseases (NCDs) in developing countries: a symposium report. *Globalization and Health*.

[10] Cohen D. J., Reynolds M. R. (2008). Interpreting the results of cost-effectiveness studies. *Journal of the American College of Cardiology*.

[11] WHO Package of Essential Noncommunicable (PEN) Disease Interventions for Primary Health Care in Low-Resource Settings.

[12] Li R., Zhang P., Barker L. E., Chowdhury F. M., Zhang X. (2010). Cost-effectiveness of interventions to prevent and control diabetes mellitus: a systematic review. *Diabetes Care*.

[13] Saha S., Gerdtham U.-G., Johansson P. (2010). Economic evaluation of lifestyle interventions for preventing diabetes and cardiovascular diseases. *International Journal of Environmental Research and Public Health*.

[14] Gaziano T. A., Galea G., Reddy K. S. (2007). Scaling up interventions for chronic disease prevention: the evidence. *The Lancet*.

[15] Husereau D., Drummond M., Petrou S. (2013). Consolidated Health Economic Evaluation Reporting Standards (CHEERS) statement. *The BMJ*.

[16] Herman W. H., Edelstein S. L., Ratner R. E. (2012). The 10-year cost-effectiveness of lifestyle intervention or metformin for diabetes prevention: an intent-to-treat analysis of the DPP/DPPOS. *Diabetes Care*.

[17] Herman W. H., Edelstein S. L., Ratner R. E. (2013). Effectiveness and cost-effectiveness of diabetes prevention among adherent participants. *American Journal of Managed Care*.

[18] van Wier M. F., Lakerveld J., Bot S. D. M., Chinapaw M. J. M., Nijpels G., van Tulder M. W. (2013). Economic evaluation of a lifestyle intervention in primary care to prevent type 2 diabetes mellitus and cardiovascular diseases: a randomized controlled trial. *BMC Family Practice*.

[19] Sagarra R., Costa B., Cabré J. J., Solà-Morales O., Barrio F. (2014). Lifestyle interventions for diabetes mellitus type 2 prevention. *Revista Clínica Española*.

[20] Kolu P., Raitanen J., Rissanen P., Luoto R. (2013). Cost-effectiveness of lifestyle counselling as primary prevention of gestational diabetes mellitus: findings from a cluster-randomised trial. *PLoS ONE*.

[21] Oostdam N., Bosmans J., Wouters M. G. A. J., Eekhoff E. M. W., van Mechelen W., van Poppel M. N. M. (2012). Cost-effectiveness of an exercise program during pregnancy to prevent gestational diabetes: results of an economic evaluation alongside a randomised controlled trial. *BMC Pregnancy and Childbirth*.

[22] Liu X., Li C., Gong H. (2013). An economic evaluation for prevention of diabetes mellitus in a developing country: a modelling study. *BMC Public Health*.

[23] Png M. E., Yoong J. S., Petta S. (2014). Evaluating the cost-effectiveness of lifestyle modification versus metformin therapy for the prevention of diabetes. *PLoS ONE*.

[24] Bertram M. Y., Lim S. S., Barendregt J. J., Vos T. (2010). Assessing the cost-effectiveness of drug and lifestyle intervention following opportunistic screening for pre-diabetes in primary care. *Diabetologia*.

[25] Mortaz S., Wessman C., Duncan R., Gray R., Badawi A. (2012). Impact of screening and early detection of impaired fasting glucose tolerance and type 2 diabetes in Canada: a Markov model simulation. *Clinicoecon and Outcomes Research*.

[26] Johansson P., Östenson C.-G., Hilding A. M., Andersson C., Rehnberg C., Tillgren P. (2009). A cost-effectiveness analysis of a community-based diabetes prevention program in Sweden. *International Journal of Technology Assessment in Health Care*.

[27] Neumann A., Schwarz P., Lindholm L. (2011). Estimating the cost-effectiveness of lifestyle intervention programmes to prevent diabetes based on an example from Germany: Markov modelling. *Cost Effectiveness and Resource Allocation*.

[28] Palmer A. J., Tucker D. M. D. (2012). Cost and clinical implications of diabetes prevention in an Australian setting: a long-term modeling analysis. *Primary Care Diabetes*.

[29] Smith K. J., Hsu H. E., Roberts M. S. (2010). Cost-effectiveness analysis of efforts to reduce risk of type 2 diabetes and cardiovascular disease in southwestern Pennsylvania, 2005–2007. *Preventing Chronic Disease*.

[30] Tsai A. G., Wadden T. A., Volger S. (2013). Cost-effectiveness of a primary care intervention to treat obesity. *International Journal of Obesity*.

[31] Cobiac L., Vos T., Veerman L. (2010). Cost-effectiveness of weight watchers and the lighten up to a healthy lifestyle program. *Australian and New Zealand Journal of Public Health*.

[32] Miners A., Harris J., Felix L., Murray E., Michie S., Edwards P. (2012). An economic evaluation of adaptive e-learning devices to promote weight loss via dietary change for people with obesity. *BMC Health Services Research*.

[33] Forster M., Veerman J. L., Barendregt J. J., Vos T. (2011). Cost-effectiveness of diet and exercise interventions to reduce overweight and obesity. *International Journal of Obesity*.

[34] Lewis L., Taylor M., Broom J., Johnston K. L. (2014). The cost-effectiveness of the Lighter Life weight management programme as an intervention for obesity in England. *Clinical Obesity*.

[35] Anokye N. K., Trueman P., Green C., Pavey T. G., Hillsdon M., Taylor R. S. (2011). The cost-effectiveness of exercise referral schemes. *BMC Public Health*.

[36] Drummond M. F., Jefferson T. O. (1996). Guidelines for authors and peer reviewers of economic submissions to the BMJ. The BMJ Economic Evaluation Working Party. *British Medical Journal*.

[37] Knowler W. C., Fowler S. E., Hamman R. F. (2009). 10-Year follow-up of diabetes incidence and weight loss in the Diabetes Prevention Program Outcomes study. *The Lancet*.

[38] Tuomilehto J., Lindström J., Eriksson J. G. (2001). Prevention of type 2 diabetes mellitus by changes in lifestyle among subjects with impaired glucose tolerance. *The New England Journal of Medicine*.

[39] Li G., Zhang P., Wang J. (2008). The long-term effect of lifestyle interventions to prevent diabetes in the China Da Qing Diabetes Prevention Study: a 20-year follow-up study. *The Lancet*.

[40] NICE (2006). *Modelling the Cost Effectiveness of Physical Activity Interventions*.

[41] Johns B., Adam T., Evans D. B. (2006). Enhancing the comparability of costing methods: cross-country variability in the prices of non-traded inputs to health programmes. *Cost Effectiveness and Resource Allocation*.

[42] Walker D., Fox-Rushby J. A. (2001). Allowing for uncertainty in economic evaluations: qualitative sensitivity analysis. *Health Policy and Planning*.

[43] Brisson M., Edmunds W. J. (2006). Impact of model, methodological, and parameter uncertainty in the economic analysis of vaccination programs. *Medical Decision Making*.

[44] Goldney R. D., Phillips P. J., Fisher L. J., Wilson D. H. (2004). Diabetes, depression, and quality of life: a population study. *Diabetes Care*.

[45] McDonough C. M., Tosteson A. N. A. (2007). Measuring preferences for cost-utility analysis: how choice of method may influence decision-making. *PharmacoEconomics*.

[46] Echouffo-Tcheugui J.-B., Kengne A.-P. (2012). A United Nation high level meeting on chronic non-communicable diseases: utility for Africa?. *Pan African Medical Journal*.

[47] Drummond M. F., Sculpher M. J., Torrance G. W., O'Brien J. B., Stoddart L. G. (2005). *Methods for the Economic Evaluation of Health Care Programmes*.

[48] ACMTS (2006). *Lignes Directrices de l'Évaluations Economique des Techonologies de Santé au Canada*.

[49] Sutton L., Karan A., Mahal A. (2014). Evidence for cost-effectiveness of lifestyle primary preventions for cardiovascular disease in the Asia-Pacific Region: a systematic review. *Globalization and Health*.

[50] Echouffo-Tcheugui J. B., Ali M. K., Griffin S. J., Narayan K. M. V. (2011). Screening for type 2 diabetes and dysglycemia. *Epidemiologic Reviews*.

[51] Eddy D. M. (2006). Accuracy versus transparency in pharmacoeconomic modelling: finding the right balance. *PharmacoEconomics*.

[52] Philips Z., Ginnelly L., Sculpher M. (2004). Review of guidelines for good practice in decision-analytic modelling in health technology assessment. *Health Technology Assessment*.

[53] Kahn R., Davidson M. B. (2014). The reality of type 2 diabetes prevention. *Diabetes Care*.

[54] Wareham N. J. (2015). Mind the gap: efficacy versus effectiveness of lifestyle interventions to prevent diabetes. *The Lancet Diabetes & Endocrinology*.

[55] Jackson S. L., Long Q., Rhee M. K. (2015). Weight loss and incidence of diabetes with the Veterans Health Administration MOVE! lifestyle change programme: an observational study. *The Lancet Diabetes & Endocrinology*.

[56] Whittemore R. (2011). A systematic review of the translational research on the Diabetes Prevention Program. *Translational Behavioral Medicine*.

